# Arecoline, a New Remedy against Acute Founder

**Published:** 1896-07

**Authors:** 


ARECOLINE, A NEW REMEDY AGAINST ACUTE FOUNDER. Fröhner used this remedy in 12 cases of acute founder with good effect. He says the symptoms disappear rapidly, even in patients which had been bled without result. Arecoline is said to act in a similar way to pilocarpine and bleeding, but more powerfully, and, moreover, is cheaper. The dose is 0.8 to 0.10 centigrams hypodermically injected, repeated daily as long as it seems necessary. Fröhner never administered it longer than seven days. According to him, this drug causes the distribution of the blood in the organism and stimulates the action of the salivary, intestinal, and sweat glands, and brings about a depletion of the system.—*Monatsheft für prak. Thierheilkunde*, vol. i., copied in *Tijdschrift voor Veeartsenijkunde*, Jan. 1896.

